# Extraction optimization of medicinally important metabolites from *Datura innoxia* Mill.: an *in vitro* biological and phytochemical investigation

**DOI:** 10.1186/s12906-015-0891-1

**Published:** 2015-10-19

**Authors:** Humaira Fatima, Komal Khan, Muhammad Zia, Tofeeq Ur-Rehman, Bushra Mirza, Ihsan-ul Haq

**Affiliations:** Department of Pharmacy, Faculty of Biological Sciences, Quaid-i-Azam University, Islamabad, 45320 Pakistan; Department of Biotechnology, Faculty of Biological Sciences, Quaid-i-Azam University, Islamabad, 45320 Pakistan; Department of Biochemistry, Faculty of Biological Sciences, Quaid-i-Azam University, Islamabad, 45320 Pakistan

**Keywords:** *Datura innoxia*, Cytotoxicity, Protein kinase inhibition, THP-1 leukemia cell line

## Abstract

**Background:**

The present study aims to probe the impact of polarity dependent extraction efficiency variation on pharmacological spectrum of *Datura innoxia* Mill. in order to reconnoiter its underexplored therapeutic potential.

**Methods:**

A range of solvent extracts was subjected to phytochemical and biological assays to find the most proficient solvent system and plant part for each type of bioactivity. Total phenolic and flavonoid contents were determined colorimetrically and specific polyphenols were quantified by HPLC-DAD analysis. The samples were biologically evaluated by employing multimode antioxidant, cytotoxic, protein kinase inhibition and antimicrobial assays.

**Results:**

Among all the solvents used, maximum percent extract recovery (33.28 %) was obtained in aqueous leaf extract. The highest amount of gallic acid equivalent phenolic and quercetin equivalent flavonoid content was obtained in the distilled water and ethyl acetate-ethanol extracts of leaf i.e., 29.91 ± 0.12 and 15.68 ± 0.18 mg/g dry weight (DW) respectively. Reverse phase HPLC-DAD based quantification revealed the presence of significant amounts of catechin, caffiec acid, apigenin and rutin ranging from 0.16 to 5.41 mg/g DW. Highest DPPH radical scavenging activity (IC_50_ = 16.14 μg/ml) was displayed by the ethyl acetate-acetone stem extract. Maximum total antioxidant capacity and reducing power potential were recorded in the aqueous leaf and ethyl acetate stem extracts i.e., 46.98 ± 0.24 and 15.35 ± 0.61 mg ascorbic acid equivalent/g DW respectively. Cytotoxicity against brine shrimps categorized 25 % of the leaf, 16 % of the stem and 8.3 % of the fruit extracts as highly potent (LC_50_ ≤ 100 μg/ml). Significant cytotoxicity against human leukemia (THP-1) cell line was exhibited by the chloroform and n-hexane fruit extracts with IC_50_ 4.52 and 3.49 μg/ml respectively. Ethyl acetate and methanol-chloroform extracts of leaf and stem exhibited conspicuous protein kinase inhibitory activity against *Streptomyces 85E* strain with 22 mm bald phenotype. A noteworthy antimicrobial activity was exhibited by leaf extracts against *Micrococcus luteus* and n-hexane fruit extract against *Aspergillus niger* (MIC 3.70 and 12.5 μg/ml respectively).

**Conclusion:**

Multiple solvent system is a crucial variable to retrieve pharmacological potential of medicinal plants and *D. innoxia* can be envisaged as a novel source of natural antioxidants, antimicrobials and anticancer compounds.

## Background

Natural products from medicinal plants, either as pure compounds or standardized extracts provide indefinite prospects for new drug leads because of the unmatched availability of chemical diversity. Modernization of ethnomedicinal plant remedies through standardization and quality control is a key factor that will govern their widespread acceptance by the international community. The chemo-diversity and cure all potential of these herbal cock-tails accentuate the role of analysis using ethnopharmacological approach as fundamental [1]. Although the bulk of current knowledge of ethnomedicinal plants has led to countless improvements in health care but unfortunately with the prompt industrialization and loss of ethnic cultures some of this information will no doubt disappear. The validation of ethno botanical data from the under explored folk plant remedies undoubtedly represent an inexhaustible reservoir of novel molecules and may lead to innovative strategies for new drug discovery. One such ethno medicinally important plant is *Datura innoxia* Mill. (Solanaceae), the native range of which appears to be China, Mexico, United States, Caribbean Islands and Asia. It is acknowledged with a common name of Thorn-Apple, Downy Thorn-Apple and Indian-Apple. It is a low growing, spreading perennial with hairy 2–5 in. leaves, white flowers and a spiny fruit. From ancient times continuing to the present, its seeds are used in shamanistic rituals as a path to enlightenment [2]. The plant conquers a very special place in Ayurveda since all plant parts namely flowers, leaves, root, stem, fruit and seeds have been meritoriously employed for a range of treatments such as insanity, rabies, leprosy, etc. However, the indiscriminate use of its extract may cause acute poisoning, delirium and can even lead to death. The active principles in *D. innoxia* include atropine, scopolamine hyoscyamine, withanolides (lactones) and other tropanes. A comparative analysis of the different seed extracts of various *Datura* species for their free radical scavenging capacity towards the stable DPPH radical revealed that *D. innoxia* has the strongest antioxidant potential [3]. Recently a new withanolide, dinoxin B, isolated from the methanol extract of *D. innoxia* leaves demonstrated a sub-micromolar IC_50_ values against multiple human cancer cell lines [4].

An exhaustive review of literature led us to the conclusion that the scientific data about the complete pharmacological spectrum of *D. innoxia* is limited. There is no organized study yet conducted involving a battery of bioassays on individual plant parts that could demonstrate a varied pharmacological potential of this ethnomedicinally important plant. The present study has employed a wide range of extraction solvent polarity as a variable to demonstrate and correlate its effects on extraction efficiency and bioactivity. To the best of our knowledge this is the first report highlighting plant’s potential for the inhibition of various protein kinases and its cytotoxic activity against human monocytic leukemia THP-1 cell lines. Since biological activity is the leading thread of ethnopharmacological approach and chemo-profiling the distinctive fingerprints for individual plant parts could be very beneficial for the development of uniform standardization tools. The present work is premeditated to evaluate the antioxidant, antifungal, antibacterial and cytotoxic potential of leaf, stem and fruit of *D. innoxia* in a scientific manner by utilizing multipolarity extraction system.

## Methods

### Plant material collection and identification

The plant material was collected in September 2013 from Quaid-i-Azam University Islamabad. Identification of the field collected plant was authenticated as *D. innoxia* by Prof. Dr. Rizwana Aleem Qureshi, Department of Plant Sciences, Faculty of Biological Sciences, Quaid-i-Azam University Islamabad, Pakistan. Dried voucher specimen was archived in the Herbarium of medicinal plants, Quaid-i-Azam University Islamabad under herbarium number PHM-487.

### Solvents and reagents

All reagents and solvents used in the present study were of analytical grade. Solvents for the extraction: ethanol, methanol, ethyl acetate, chloroform, acetone, n-hexane and dimethylsulfoxide (DMSO) were purchased from Merck (Darmstadt, Germany). Folin–Ciocalteu reagent, 2, 2-diphenyl, 1-picrylhydrazine (DPPH) was acquired from Sigma–Aldrich (Steinheim, Germany). All other chemicals (analytical grade) and reagents i.e., sodium hydroxide, ferrous chloride, aluminum chloride, potassium dihydrogen phosphate, dipotassium hydrogen phosphate, quercetin, gallic acid, ascorbic acid, caffeic acid, rutin, kaempferol, (+)-catechin and myricetin used in this study were purchased from Merck (Darmstadt, Germany), unless stated otherwise.

### Preparation of crude extract

The plant was washed thoroughly under running water to remove contamination and was shade dried with active ventilation at ambient temperature for three weeks. The dried stem, fruit and leaves were ground separately to fine powder using electric knife mill and stored in air-tight containers. The powdered plant parts were subjected to extraction by sonication aided maceration using analytical grade solvents i.e., n-hexane (Nh), chloroform (C), acetone (A), ethyl acetate + acetone (EthA), ethyl acetate (Eth), ethanol + chloroform (EC), methanol + chloroform (MC), ethyl acetate + ethanol (EthE), methanol + ethyl acetate (MEth), methanol (M), ethanol (E) and distilled water (D). A ratio of 1:1 was used for preparing extraction solvent having various solvent combinations as described above. Accurately weighed (40 g) plant material was macerated with 400 ml solvent in 1000 ml Erlenmeyer flask and was subjected to maceration for 24 h at room temperature followed by sonication (ultrasonic bath, room temperature, 30 min). The marc was extracted twice using same procedure and the extracts were combined which were then filtered through muslin cloth followed by filtration through Whatmann No. 1 filter paper. The extracts were then concentrated with vacuum evaporation in rotary evaporator (Buchi, Switzerland) and dried in vacuum oven (Yamato, Japan) at 45 °C to obtain final crude extract and the experiment was run in triplicate.

### Percent extract recovery

The dried extracts were weighed to calculate the percent recovery of crude extract by the following formula.

$$ \%\ \mathrm{Extract}\ \mathrm{recovery}\kern0.5em =\kern0.5em \left(\mathrm{A}/\mathrm{B}\right)\times 100 $$

A = Total weight of dried extract obtained after drying.

B = Total weight of ground plant material taken for each extraction.

### Phytochemical analysis

#### Determination of total phenolic content

The total phenolic contents were estimated according to slightly modified procedure as described previously using Folin–Ciocalteu reagent [5, 6]. An aliquot of 20 μl from 4 mg/ml DMSO stock solution of each test sample was transferred in respective well of 96 well plate followed by addition of 90 μl of Folin–Ciocalteu reagent. The plate was incubated for 5 min after which 90 μl of sodium carbonate was added to the reaction mixture. Gallic acid was used as standard and absorbance of each reaction mixture was taken after triplicate performance at 630 nm using microplate reader (Biotech USA, microplate reader Elx 800). A calibration curve (y = 0.0136x + 0.0845, R^2^ = 0.9861) was obtained in parallel under the same operating conditions using gallic acid (6.25–50 μg/ml) as a positive control and the correlation was found to be significant at 0.05 level. The assay was performed in triplicate and the results are expressed as mg gallic acid equivalent per gram dry weight (mg GAE/g DW).

#### Determination of total flavonoid content

For total flavonoid content determination, aluminum chloride colorimetric method was employed with slight modifications according to system suitability [7, 8]. The crude extracts (20μl of 4.0 mg/ml DMSO) were transferred to each well of 96 well plate. Subsequently, 10 μl each of 10 % aluminum chloride and 1.0 M potassium acetate was added followed by the addition of 160 μl of distilled water. The resulting mixture was kept at room temperature for 30 min. Then absorbance of the plate was measured at 415 nm using microplate reader. The calibration curve was drawn by using quercetin as standard at final concentrations of 0, 2.5, 5, 10, 20, 40 μg/ml, the assay was performed in triplicate and the resultant flavonoid content was documented in mg equivalents of quercetin per gram of plant dry weight (mg QE/g DW). The equation obtained for the calibration curve of quercetin was y = 0.0268x + 0.00764 (R^2^ = 0.9986) and the correlation was found to be significant at 0.05 level.

#### HPLC-DAD quantitative analysis

High performance liquid chromatography was performed by using Agilent Chem station Rev. B.02-01-SR1 (260) and Agilent 1200 series binary gradient pump coupled with diode array detector (DAD; Agilent technologies, Germany). Reverse phase chromatographic analysis was carried out with a Zorbex-C8 analytical column (4.6 × 250 mm, 5 μm particle size, Agilent, USA), injection volume 20 μl and the gradient elution was conducted according to the method previously described with minor modifications [7]. Mobile phase consisted of acetonitrile-methanol–water-acetic acid in a ratio of 5:10:85:1 (solvent A) and acetonitrile-methanol-acetic acid in a ratio of 40:60:1(solvent B). Gradient method was 0–20 min for 0–50 % B, 20–25 min for 50–100 % B and then isocratic 100 % B till 30 min. Flow rate was maintained at 1 ml/min. Stock solutions of various phenolic standards i.e., phenolic acid (gallic acid), flavan-3-ol (catechin), flavonol flavonoids (quercetin, myricetin, kaempferol), hydroxycinnamate (caffeic acid), flavone aglycone (apigenin) and flavonol glycoside (rutin) were prepared in methanol and subsequently diluted to get a final concentration of 10, 20, 50, 100, 200 μg/ml of methanol. The data for peak area versus standard concentration was used to construct the calibration curve, the correlations were found to be significant at 0.05 level, results of which are summarized in Table 2. The respective limit of detection (LOD) and limit of quantification (LOQ) as determined by linear regression analysis of the calibration curve were calculated by using the expression 3.3 * (σ/b) and 10 * (σ/b) respectively where;

σ = Standard deviation of response

b = Slope of calibration curve

Prior to use, standard solutions, samples and mobile phases were all degassed and filtered through 0.45 μm membrane filter (Millipore). The absorption of samples was recorded at 257nm (rutin), 279 nm (gallic acid, catechin), 325 nm (caffeic acid, apigenin) and 368 nm (myricetin, quercetin and kaempferol).

Chromatographic operation was carried out at ambient temperature and in triplicate. Before starting the next analysis column was reconditioned for 10 min and the results were expressed as mg/g DW. Comparison of retention time and UV absorption spectra of extracts with those of standards was done for the identification of compounds.

### Biological evaluation

#### Radical scavenging activity-DPPH assay

The antioxidant potential of the crude extracts was gauged by monitoring its capacity to quench the stable 2, 2-diphenyl 1-picrylhydrazyl (DPPH) free radical [5, 9]. Spectrophotometric analysis was used to measure the percent radical scavenging capacity (%RSA) and to determine the corresponding 50 % inhibitory (scavenging) concentration (SC_50_). The DPPH quenching ability was expressed as IC_50_, the concentration required to inhibit radical formation by 50 %. Four different dilutions of each test extract (20 μl), to obtain final concentrations of 200, 66.66, 22.22 and 7.406 μg/ml, were mixed with 180 μl of 9.2 mg/100 ml methanol DPPH solution in 96 well plates. The absorbance was measured at 517 nm using microplate reader after 30 min of reaction at 37 °C. Scavenging activity in percent (%RSA) was calculated by using the equation:

$$ \%\mathrm{R}\mathrm{S}\mathrm{A} = \left(1\hbox{-} {\mathrm{Ab}}_{\mathrm{s}}/{\mathrm{Ab}}_{\mathrm{c}}\right)\times 100 $$

Where Ab_s_ is the absorbance of DPPH solution with sample, whereas Ab_c_ is the absorbance of negative control containing the reagent except the sample. Ascorbic acid was used as positive control and the assay was performed in triplicate.

#### Total antioxidant capacity estimation by phosphomolybdenum based assay

Phosphomolybdenum based total antioxidant capacity of the extracts was estimated concisely by mixing 0.1 ml of each test extract (4 mg/ml DMSO) and positive control (ascorbic acid, 4 mg/ml) with 1 ml of reagent (0.6 M sulphuric acid, 28 mM sodium phosphate and 4 mM ammonium molybdate). A typical blank solution consisted of 1 ml of reagent solution and the appropriate volume of the same solvent used for each sample. All tubes were incubated in a boiling water bath for 90 min at 95 °C. After the samples had been cooled to room temperature, the absorbance of the each sample solution was measured at 695 nm against the blank using a PDA spectrophotometer (8354 Agilent Technologies, Germany). The experiment was performed in triplicate. The antioxidant activity was expressed as the number of mg equivalents of ascorbic acid per gram of dry plant weight i.e., mg AAE/g DW [7, 10].

#### Total reducing power estimation by potassium ferricyanide colorimetric assay

The reducing power of different solvent extracts was determined in accordance with the method described previously [7, 11]. Concisely, 200 μl of each test extract (4 mg/ml DMSO) was mixed with 400 μl of phosphate buffer (0.2 mol/l, pH 6.6) and 1 % potassium ferricyanide [K_3_Fe (CN)_6_]. The mixture was then incubated at 50 °C for 20 min. A portion of trichloroacetic acid (400 μl of 10 %) was added to the mixture, which was then centrifuged at 3000 rpm at room temperature for 10 min. The upper layer of solution (500 μl) was mixed with distilled water (500 μl) and FeCl_3_ (100 μl, 0.1 %). The absorbance was measured at 700 nm and an increased absorbance of the reaction mixture indicated increased reducing power. Blank was prepared by adding 200 μl of DMSO to the aforementioned reaction mixture instead of the extract. The reducing power of each sample was expressed as mg ascorbic acid equivalent per gram plant dry weight (mg AAE/g DW) and the assay was performed as triplicate analysis.

#### Cytotoxicity assays: Brine shrimp lethality assay

A 24 h lethality test was performed in a 96 well plate against brine shrimp (*Artemia salina*) larvae according to the previously described protocol with minor modifications [5]. Eggs of test organism *Artemia salina* (Ocean 90, USA) were kept for 24–48 h hatching period in simulated sterile sea water (38 g/l supplemented with 6 mg/l dried yeast) with constant oxygen supply in a specially designed two-compartment plastic tray under illumination, providing direct light and warmth (30–32 °C). The mature phototropic nauplii were then harvested with the help of Pasteur pipette and transferred to each well of 96 well plate. Test extracts were initially tested at three graded concentrations i.e., 1000, 500 and 250 μg/ml and corresponding volume of each extract was then transferred to the wells containing sea water and shrimp larvae. Positive and negative control wells included standard doxorubicin (4 mg/ml) and DMSO respectively instead of sample. After 24 h incubation period, degree of lethality exhibited by each solvent extract was determined by counting the number of survivors and median lethal concentration (LC_50_) of the test samples with ≥ 50 % mortality, was calculated using table curve 2D v5.01 software. The whole experiment was run as triplicate analysis.

#### Cytotoxicity against THP-1 human leukemia cell line

The *in vitro* cytotoxicity evaluation of extracts was carried out by using human leukemia (THP-1) cell lines (ATCC # TIB-202) according to the previously documented protocol with slight modification according to system suitability [5]. Briefly, leukemia cells were cultured in complete growth medium [RPMI-1640 medium buffered with 2.2 g/l NaHCO_3_ and supplemented with 10 % heat inactivated fetal bovine serum (HIFBS); pH 7.4] in a carbon dioxide incubator (37 °C, 5 % CO _2_). About 190 μl of THP-1 cells at a seeding density of 5 × 10^5^ cells per ml were transferred to each well of 96-well microtiter plate. Subsequently, 10 μl of sample containing 1 % DMSO in PBS was added. Samples were tested three times at concentrations of 10, 5 and 2.5 μg/ml. The reaction plate was incubated at 37 °C in humidified CO_2_ (5 %) incubator for 72 h. Florouracil and vincristine (4 mg/ml DMSO) were used as positive standard drug in positive control wells whereas 1 % DMSO in PBS served as negative control. The number of survivors was counted using improved neubauer chamber (Marien, Germany) under a light microscope and compared with percent survival of cells in the presence of positive and negative controls and the assay was performed in triplicate. Afterwards LC_50_ was calculated by using table curve 2D v5.01 software.

#### Protein kinase inhibition assay

The protein kinase inhibition assay was performed thrice by observing hyphae formation in purified isolates of *Streptomyces* 85E strain [12]. Bacterial lawn was allowed to develop by spreading spores (mycelia fragments) of refreshed culture of *Streptomyces* on sterile plates containing minimal ISP4 medium. About 5 μl of each extract (20 mg/ml of DMSO) was loaded onto sterile 6 mm filter paper discs. The impregnated paper discs with a final concentration of 100 μg/disc were applied directly on the surface of the plates seeded with *Streptomyces* 85E. Surfactin and DMSO infused discs were included as positive and negative control respectively. The plates were then incubated at 30 °C for 72 h (time required for hyphae formation in *Streptomyces* 85E) and the results were interpreted as bald zone of inhibition around samples and controls infused discs.

### Antimicrobial assays

#### Antibacterial assay

*In vitro* antibacterial potential of the given test extracts was evaluated by agar disc diffusion method as described previously [13]. A lawn of refreshed bacterial cultures [(gram positive: *Staphylococcus aureus* ATCC-6538, *Micrococcus luteus* ATCC-10240) and gram negative (*Salmonella typhimurium* ATCC*-*14028, *Klebsiella pneumonia* ATCC-1705)] with pre-adjusted seeding density was made on nutrient agar plates. Sterile filter paper discs impregnated with 5 μl (20 mg/ml DMSO) of each test extract were placed on the seeded plates. Disc infused with Cefixime (standard antibacterial) served as positive control while DMSO infused disc was used as negative control. Following incubation at 37 °C for 24 h, the average diameter of the zone of inhibition around the sample as well as control treated discs was measured and recorded. Extracts producing an inhibition zone ≥ 10 mm in diameter were considered active and were further screened to determine minimum inhibitory concentrations (MICs) by standard three-fold microbroth dilution methodology [14]. A stock solution of each active extract was serially diluted in 96-well microtiter plate with Mueller Hinton broth to obtain a concentration ranging from 100 μg/ml to 3.70 μg/ml. A standardized inoculum for each bacterial strain was prepared so as to give inoculum size of approximately 5 × 10^4^ CFU/ml in each well. Microtiter plates were then kept at 37 °C for an overnight incubation. Following incubation, the MIC was calculated as the lowest concentration of the extract inhibiting the growth of bacterial strain by measuring OD at 600 nm and the assay was performed as triplicate analysis.

#### Antifungal assay

The antifungal potential of test extracts was evaluated as triplicate analysis by agar disc diffusion method [13]. The spores of given fungal strains [*Aspergillus fumigatus* (FCBP- 66), *Mucor* species (FCBP-0300), *Aspergillus niger* (FCBP-0198) and *Aspergillus flavus* (FCBP-0064)] were harvested in 0.02 % Tween 20 solution and their turbidity was adjusted according to McFarland 0.5 turbidity standard. Then 100 μL of each harvested fungal strain was swabbed on plates containing Sabouraud Dextrose agar. Sterile filter paper discs impregnated with 5 μl (20 mg/ml DMSO) each of test extract were placed on the seeded plates. DMSO impregnated disc was used as negative control whereas standard drug clotrimazole exhibited maximum activity with 30 ± 1.54 mm zone. Following incubation at 28 °C for 24–48 h, the average diameter (mm) of the zone of growth inhibition around the samples as well as control treated discs was measured and recorded. Extracts producing an inhibition zone ≥ 10 mm in diameter were screened to determine minimum inhibitory concentrations (MICs) at lower concentrations ranging from 50 to 3.12 μg/disc by standard disc diffusion method. MIC was calculated as the lowest concentration of the extract around which a visible zone of growth inhibition was formed.

#### Statistical analysis

The results obtained for cytotoxic, antimicrobial and phytochemical assays were analyzed statistically by one-way analysis of variance (ANOVA) followed by Tukey and Duncan’s test using the statistical package PASW Statistics 18 and *P* < 0.05, *P* < 0.01, or *P* < 0.001 was considered as significant when appropriate. Data were expressed as mean ± SD. Correlation analysis of the phytochemical activities and HPLC-DAD analysis was carried out using the correlation and regression by Microsoft Excel program.

## Results and discussion

### Effect of extraction solvent on the extract yields

Percentage of the extract recovery determined for different plant parts, using sonication followed by maceration as extraction technique is presented in Table 1. Maximum amount of extract was recovered when distilled water was used as the extraction solvent with an extract yield of 33.28 ± 0.87 %, 18.88 ± 1.18 % and 16.83 ± 0.99 % for leaf, stem and fruit respectively. It has been observed that as the polarity of extraction solvent changed from highly polar water to non-polar n-hexane, the extract yields decreased drastically. The differences in the extract yields in the present analysis might be endorsed to the different availability of extractable components, resulting from the varied chemical composition of plant metabolites. The extraction efficiency and biological activities are strongly dependent on the nature of extraction solvent polarity, due to the presence of diverse compounds of varied chemical characteristics that may or may not be soluble in a particular solvent. As extraction is the first critical step in drug discovery process from plants, therefore in our present study a wide range of extraction solvent polarities and sonication followed by maceration as extraction technique was employed. It has also been suggested from their extraction yield optimization studies of herbal material that a sample preparation method, maceration under sonication proved to be a superlative choice when considering the time/yield ratio [15].

**Table 1:** Percent extract recoveries of leaf, stem and fruit of *D. innoxia* using different extraction solvents

Extracts	% Extract recovery		
	Leaf	Stem	Fruit
Nh	0.75 ± 0.20_b_^a^	3.90 ± 0.87_a_^b^	4.05 ± 0.11_b_^a^
C	6.23 ± 0.47_c_^b^	8.83 ± 0.64_c_^b^	11.28 ± 0.10_c_^a^
A	15.07 ± 0.94_a_^c^	13.08 ± 0.22_a_^b^	5.53 ± 0.49_a_^c^
EthA	15.66 ± 0.76_a_	11.96 ± 0.62_a_^c^	2.49 ± 0.33_b_^b^
Eth	11.10 ± 0.74_b_^c^	13.62 ± 0.56_a_^b^	4.37 ± 0.48_b_^c^
EC	7.63 ± 1.66_b_^b^	6.97 ± 0.44_b_^c^	6.30 ± 0.16_a_^b^
MC	8.01 ± 0.40_b_^a^	9.18 ± 2.88_b_^c^	9.63 ± 0.71_a_^b^
EthE	11.33 ± 0.85_c_^b^	5.79 ± 0.46_c_^a^	7.90 ± 1.41_b_^a^
MEth	8.55 ± 0.73_c_	10.45 ± 1.96_c_^a^	9.38 ± 0.34_a_^c^
E	8.82 ± 0.33_b_^c^	6.16 ± 0.72_c_^c^	7.03 ± 1.94_a_^d^
M	8.79 ± 0.93_c_^b^	3.43 ± 1.45_b_^b^	11.61 ± 1.53_a_^c^
D	33.28 ± 0.87_a_^a^	18.88 ± 1.18_b_^a^	16.83 ± 0.99_b_^a^

Values (mean ± SD) are average of three samples of each plant part, analyzed individually in triplicate (*n* = 1 × 3 × 3), (*P* < 0.05); *DW* dry weight; Superscript letters within the same column indicate significant (*P* < 0.05) differences of means within the extracting solvent; Subscript letters within the same row indicate significant (*P* < 0.05) differences of means within the plant parts. *Nh* n-hexane, *C* Chloroform, *A* Acetone, *EthA* Ethyl acetate-Acetone, *Eth* Ethyl acetate, *EC* Ethanol-Chloroform, *MC* Methanol-Chloroform, *EthE* Ethyl acetate-Ethanol, *MEth* Methanol-Ethyl acetate, *E* Ethanol, *M* Methanol, *D* Distilled water

### Phytochemical analysis

#### Total phenolic content

**Fig. 1:**
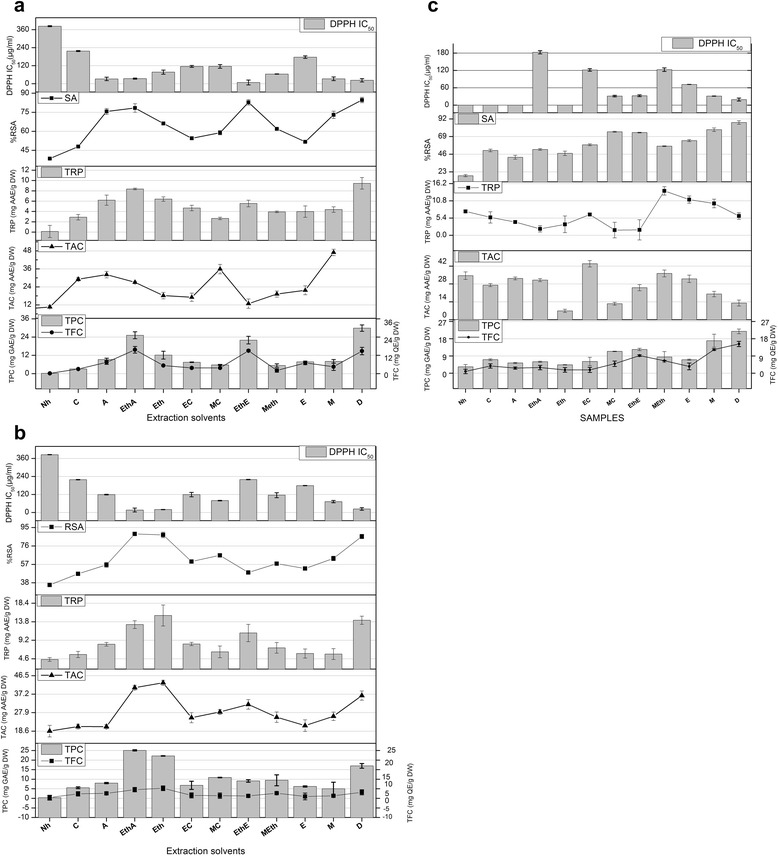
**a Leaf**: TPC (Total phenolic content mg GAE/g DW), TFC (Total flavonoid content mg QE/g DW), TAC (Total antioxidant capacity mg AAE/g DW), TRP (Reducing power mg AAE/g DW) and %RSA (radical scavenging activity) determination in different solvent extracts of *D. innoxia.* Values are presented as mean ± Standard error from triplicate investigation. Nh: n-hexane, C: Chloroform, A: Acetone, EthA: Ethyl acetate-Acetone, Eth: Ethyl acetate, EC: Ethanol-Chloroform, MC: Methanol-Chloroform, EthE: Ethyl acetate-Ethanol, MEth: Methanol-Ethyl acetate, E: Ethanol, M: Methanol, D: Distilled water. **b Stem**: TPC (Total phenolic content mg GAE/g DW), TFC (Total flavonoid content mg QE/g DW), TAC (Total antioxidant capacity mg AAE/g DW), TRP (Reducing power mg AAE/g DW) and %RSA (radical scavenging activity) determination in different solvent extracts of *D. innoxia.* Values are presented as mean ± Standard error from triplicate investigation. Nh: n-hexane, C: Chloroform, A: Acetone, EthA: Ethyl acetate-Acetone, Eth: Ethyl acetate, EC: Ethanol-Chloroform, MC: Methanol-Chloroform, EthE: Ethyl acetate-Ethanol, MEth: Methanol-Ethyl acetate, E: Ethanol, M: Methanol, D: Distilled water. **c Fruit**: TPC (Total phenolic content mg GAE/g DW), TFC (Total flavonoid content mg QE/g DW), TAC (Total antioxidant capacity mg AAE/g DW), TRP (Reducing power mg AAE/g DW) and %RSA (radical scavenging activity) determination in different solvent extracts of *D. innoxia.* Values are presented as mean ± Standard error from triplicate investigation. Nh: n-hexane, C: Chloroform, A: Acetone, EthA: Ethyl acetate-Acetone, Eth: Ethyl acetate, EC: Ethanol-Chloroform, MC: Methanol-Chloroform, EthE: Ethyl acetate-Ethanol, MEth: Methanol-Ethyl acetate, E: Ethanol, M: Methanol, D: Distilled water

The total phenolic content of leaf, stem and fruit of *D. innoxia* are presented in Fig. 1. In our study the highest content of gallic acid equivalent phenols i.e., 29.91 ± 0.12 and 21.86 ± 0.45 mg/g DW was present in the aqueous extract of leaf and fruit extracts respectively while in case of stem, it was highest in the ethyl acetate-acetone extract (25.06 ± 0.45 mg GAE/g DW). Among leaf extracts the phenolic content decreased in accordance with the following order of extraction solvent polarity; D ˃ EthA ˃ EthE ˃ Eth ˃ A ˃ M ˃ E ˃ EC ˃ MC ˃ MEth ˃ C ˃ Nh. The stem extracts displayed a pattern of decreasing phenolic content in the following order: EthA ˃ D ˃ Eth ˃ MC ˃ MEth ˃ EthE ˃ A ˃ EC ˃ E ˃ C ˃ M ˃ Nh while it was D ˃ M ˃ EthE ˃ MC ˃ MEth ˃ C ˃ E ˃ EC ˃ Eth ˃ EthA ˃ A ˃ Nh in case of fruit extracts. The total phenols in different plant parts ranged from 29.91 ± 0.12 mg GAE/g DW for the highly polar aqueous to 2.5 ± 0.12 mg GAE/g DW non-polar n-hexane, which is in agreement with the results of previous studies that quantification of phenolic compounds in plant extract is influenced by the chemical nature of the extraction solvent (Kumaran et al. [16]). The plant phenolics having antioxidant properties have an important role in combating oxidative stress, cytotoxicity and cell death by scavenging free radicals or chelating trace elements thereby strengthening the antioxidant defenses [17]. In most of the medicinal plants, phenolic and polyphenolic compounds such as flavonoids, phenolic acids and tannins are the major contributors to the antioxidant activity.

#### Total flavonoid content

The total flavonoid content of leaf, stem and fruit in terms of mg quercetin equivalent per gram dry weight are presented in Fig. 1. Among all the leaf extracts the highest flavonoid content of 15.68 ± 0.18 mg QE/g DW was recorded in the ethyl acetate-acetone extract followed by D ˃ A ˃ E ˃ Eth ˃ M ˃ MC = E ˃ EC ˃ C ˃ MEth ˃ Nh while in case of stem extracts, the maximum flavonoids were quantified in the ethyl acetate extract yielding about 5.29 ± 1.22 mg QE/g DW. The aqueous fruit extract unveiled the highest total flavonoid content of 15.28 ± 1.132 mg QE/g DW as compared to other plant parts analyzed. A positive correlation (correlation coefficient; R^2^ = 0.9137 for leaf, 0.8026 for stem and 0.8999 for fruit) was found to be present between the phenolic and flavonoid contents suggesting that the antioxidant potential of phenols might be attributed to the presence of flavonoids.

#### HPLC-DAD quantitative analysis

**Fig. 2:**
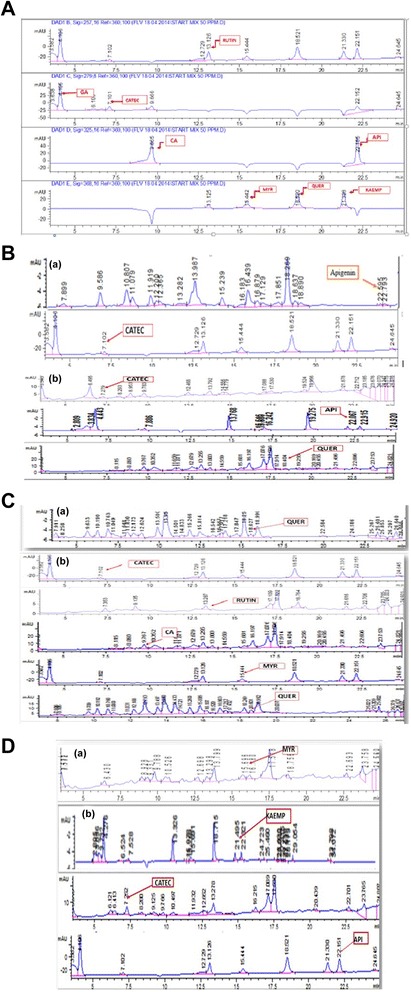
**a** Chromatograms of standard phenols. **b** Chromatograms of compounds detected in (a) MC: Methanol-Chloroform and (b) M: Methanol extracts of *D. innoxia* leaf. **c** Chromatograms of compounds detected in (a) A: Acetone and (b) D: Distilled water extracts of *D. innoxia* stem. **d** Chromatograms of compounds detected in (a) A: Acetone and (b) E: Ethanol extracts of *D. innoxia* fruit

Reverse phase HPLC-DAD based profiling was used for quantitative analysis of selected plant phenolics and the chromatographic finger printing was done by comparing the retention time and UV spectra of reference compounds with those of the test sample, the results of which are summarized in Tables 2, 3 and 4. A significant amount of catechin, myricetin, quercetin, rutin and caffeic acid were quantified in some of the analyzed extracts. Among the leaf extracts a substantial amount of catechin and apigenin was present in the methanol (5.41 and 2.11 μg/mg DW respectively) and methanol-chloroform (1.28 and 1.78 mg/g DW respectively) extract. Significant amount of catechin and apigenin were also quantified in ethanolic extract of fruit (2.65 and 2.46 mg/g DW respectively). The presence of all these plant metabolites draw a parallel correlation of plants potential with their known bioactivities e.g., rutin is one of the phenolic compounds found in the invasive plant species and contributes to its antibacterial and antioxidant properties, caffeic acid outperformed the other antioxidants in reducing aflatoxin production by more than 95 % and apigenin induces autophagy in leukemia cells, which may support a plant’s possible chemopreventive and anticancer role [18]. The chromatograms of standards as well as compounds detected in various plant parts are presented in Fig. 2.

**Table 2:** Calibration curve parameters, limit of detection (LOD), limit of quantification (LOQ) for the standards

Standard	Retention time (min)	Calibration curve equation	Correlation coefficient (r^2^)	LOD (μg/ml)	LOQ (μg/ml)
Gallic Acid	4.19	y = 24.857x – 45.174	0.9979	7.23	21.93
Catechin	7.10	y = 7.9854x – 17.565	0.9995	3.52	10.69
Caffiec Acid	9.66	y = 26.097x + 95.435	0.9924	13.67	41.45
Rutin	3.12	y = 8.3367x + 22.217	0.9966	9.91	27.85
Myrisitin	15.44	y = 5.2278x – 6.3043	0.9988	5.40	16.37
Quercitin	18.52	y = 12.21x – 20.348	0.9978	7.35	22.29
Kaempherol	21.328	y = 9.9944x + 15.261	0.9998	2.30	6.99
Apigenin	22.156	y = 18.111x + 25.565	0.997	5.04	15.29

**Table 3:** Chemical profiling of different solvent extracts of *D. innoxia* using HPLC-DAD

Extracts	Polyphenols (μg/mg DW)							
	Phenolic acid	Flavonol glycoside	Hydroxy cinnamate	flavan-3-ol	Flavone aglycone	Flavonol flavonoid		
	GA	Rutin	CA	Catec	Api	Myr	Quer	Kaemp
Leaf								
Nh	--	--	--	--	--	--	--	--
C	--	--	--	--	--	--	--	--
A	--	--	--	--	--	--	--	--
EthA	--	--	--	--	--	--	--	--
Eth	--	--	--	--	--	--	--	--
EC	--	--	--	--	--	--	--	--
MC	--	--	--	1.28 ± 0.01	1.78 ± 0.02	--	--	--
EthE	--	--	--	--	--	--	--	--
MEth	--	--	--	--	--	--	--	--
E	--	--	--	--	--	--	--	--
M	--	--	--	5.41 ± 0.03	2.11 ± 0.01	--	0.84 ± 0.02	--
D	--	--	--	--	--	--	--	--
Stem								
Nh	--	--	--	--	--	--	--	--
C	--	--	--	--	--	--	--	--
A	--	--	--	--	--	--	1.33 ± 0.01	--
EthA	--	--	--	--	--	--	--	--
Eth	--	--	--	--	--	--	--	--
EC	--	--	--	--	--	--	--	--
MC	--	--	--	--	--	--	--	--
EthE	--	--	--	--	--	--	--	--
MEth	--	--	--	--	--	--	--	--
E	--	--	--	0.18 ± 0.01	0.16 ± 0.03	--	--	--
M	--	--	--	--	--	--	--	--
D	--	1.58 ± 0.02	1.69 ± 0.01	0.51 ± 0.01	--	1.67 ± 0.03	0.67 ± 0.01	--
Fruit								
Nh	--	--	--	--	--	--	--	--
C	--	--	--	--	--	--	--	--
A	--	--	--	1.75 ± 0.01	--	--	--	--
EthA	--	--	--	--	--	--	--	--
Eth	--	--	--	--	--	--	--	--
EC	--	--	--	--	--	--	--	--
MC	--	--	--	--	--	--	--	--
EthE	--	--	--	--	--	--	--	--
MEth	--	--	--	--	--	--	--	--
E	--	--	--	2.65 ± 0.02	2.46 ± 0.02	1.74 ± 0.01	--	0.85 ± 0.01
M	--	--	--	--	--	--	--	--
D	--	--	--	--	--	--	--	--

--: not detected, *GA* gallic acid, *CA* caffeic acid, *Catec* catechin, *Api* apigenin, *Myr* myrecetin, *Quer* quercetin, *Kaemp* kaempferol

**Table 4:** Retention time (RT) in minutes and detection wavelength (λ) in nanometers of detected compounds in different solvent extracts of leaf, stem and fruit of *D. innoxia*

Extracts	Polyphenols (μg/mg DW)													
	Rutin		CA		Catec		Api		Myr		Quer	Kaemp		
	RT (min)	λ (nm)	RT (min)	λ (nm)	RT (min)	λ (nm)	RT (min)	λ (nm)	RT (min)	λ (nm)	RT (min)	λ (nm)	RT (min)	λ (nm)
Leaf														
MC	--	--	--	--	7.10	279	22.06	325	--	--	--	--	--	--
M	--	--	--	--	7.27	279	22.07	325	--	--	18.40	368	--	--
Stem														
A	--	--	--		--		--		--	--	18.62	368		
E					7.21	279	22.21	325	--	--	--	--	--	--
D	13.28	257	9.76	325	7.12	279	--		15.44	368	--	--	--	--
Fruit														
A	--	--	--	--	--	--	--	--	--	--	18.42	368	--	--
E	--	--	--	--	7.25	279	22.15	325	15.69	368	--	--	21.49	368

--: not detected, *GA* gallic acid, *CA* caffeic acid, *Catec* catechin, *Api* apigenin, *Myr* myrecetin, *Quer* quercetin, *Kaemp* kaempferol

### Biological evaluation

#### DPPH radical scavenging activity

The percent free radical scavenging activity (%RSA) of the crude extracts was assessed by the discoloration of the methanolic solution of DPPH, the results of which are summarized in Fig. 1. This method depends on the reduction of the purple colored DPPH, by accepting electron or hydrogen radical from donor antioxidant, to a stable yellow-colored diphenyl picrylhydrazine molecule (Haq et al. [5]). The best antioxidant activity in DPPH assay was demonstrated by the aqueous extract of leaf (IC_50_ = 22.83 μg/ml) while it was lowest for n-hexane (IC_50_ = 383.31 μg/ml). Ethyl acetate-acetone extract of stem exhibited highest scavenging activity with an IC_50_ of 16.14 μg/ml followed by ethyl acetate (IC_50_ = 19.34 μg/ml) and aqueous (IC_50_ = 23.22 μg/ml) extracts. Among all the fruit extracts the most potent radical scavenging potential was exhibited by the aqueous extract with an IC_50_ of 19.22 μg/ml.

The relationship between total contents of polyphenols and the free radical scavenging capacity as studied by many authors demonstrated that a linear correlation exists between them [19]. In our present study a significant correlation was also found between the radical scavenging capability and the total phenolic content (leaf: R^2^ = 0.7094, stem: R^2^ = 0.7739, fruit: R^2^ = 0.8243). Therefore it can be inferred that the various polyphenols in *D. innoxia* extracts are largely responsible for the radical scavenging mediated antioxidant activity.

#### Total antioxidant capacity

The total antioxidant capacity (TAC) of various leaf, stem and fruit extracts are summarized in Fig. 1. The method relies on the reduction of Mo (VI) to Mo (V) by the antioxidant mediators and the consequent formation of a green colored phosphate/Mo (V) complex with a maximal absorption at 695 nm (Jafri et al. [7]). Maximum TAC was displayed by the aqueous extract of the leaf (46.98 ± 0.14 mg AAE/g DW), ethyl acetate extract of the stem (42.90 ± 1.25 mg AAE/g DW) and ethanol-chloroform extract of the fruit (43.86 ± 1.18 mg AAE/g DW). Oxidation is a mandatory natural phenomenon in biological systems resulting in the generation of highly reactive hydroxyl and a peroxyl radical. Antioxidants quench these radicals, the inadequate endogenous or exogenous supply of which may cause damage to DNA, proteins and polyunsaturated fatty acid residues of cell membrane phospholipids and may lead to pathological effects such as vascular diseases and cancer [20]. The correlation between total phenolic content and antioxidant capacity was determined and it was found to be linear with an excellent correlation coefficient, R^2^ of 0.7821 (leaf), 0.8544 (stem), 0.8702 (fruit) respectively. These results are in agreement with the previous studies which showed that high phenolic content increases the antioxidant activity [1, 20].

#### Reducing power

Figure 1 shows the reductive power of various solvent extracts of *D. innoxia*. In our present study, the maximum extraction efficiency in terms of highest reducing power was achieved in the aqueous extract of leaf (9.46 ± 1.12 mg AAE/g DW), ethyl acetate extract of stem (15.35 ± 0.61 mg AAE/g DW) and methanol-acetone extract (13.90 ± 0.87 mg AAE/g DW) of fruit. The reducing properties are generally allied with the presence of reductones which have been linked to the antioxidant action through breakage of the free radical chain by donating a hydrogen atom, therefore a direct correlation have been observed between the antioxidant capacity and reducing power of certain plant extracts [20]. In our present exploration a positive correlation was also found to exist between the reducing power and antioxidant potential of all the extracts with excellent correlation coefficients (R^2^ = 0.8058, 0.8497, 0.80121 for leaf, stem and fruit respectively) significant at the *P* = 0.05 level.

### Cytotoxicity assays

#### Brine shrimp lethality assay

Cytotoxicity potential of the plant was tested against brine shrimp (*Artemia salina*) larvae to reveal its lethality profile. Out of a total of 36 organic extracts screened for cytotoxic activity against brine shrimp larvae, 25 % of the leaf, 16 % of the stem and 8.3 % of the fruit extracts demonstrated activity at or below 100 μg/ml and were categorized as highly toxic. The remaining 75 % of the leaf, 84 % of the stem and 91.7 % of the fruit extracts had LC_50_ values ≤ 250 μg/ml and were categorized as toxic. The results from screening of the organic extracts of different plant organs against *A. salina* larvae are shown in Table 5. The positive control, doxorubicin demonstrated an LC_50_ value 5.93 μg/ml. Among all the individual plant part extracts, methanol-chloroform was found to be the most toxic exhibiting an LC_50_ of 85.94 μg/ml for leaf and stem while 54.07 μg/ml for fruit; indicating that a moderately polar extraction solvent would be the best choice for the isolation of such compounds rather than a highly polar or non-polar solvent. The degree of lethality was found to be directly proportional to the concentration of the extract. The brine shrimp lethality assay is well-thought-out as a suitable tool for primary evaluation of toxicity. It has also been proposed for screening pharmacological activities in plant extracts and the toxicity results can be correlated with their documented ethno-pharmacological role. These tests are usually carried to draw extrapolations on the safety of the plant extracts and further to illustrate trends of their biological activities [21]. In bioactivity evaluation of plant extracts by brine shrimp bioassay, an LC_50_ value of less than 1000 μg/ml is considered to be cytotoxic. In our current study, 100 % of all the screened organic extracts demonstrated LC_50_ values < 1000 μg/ml, signifying the presence of cytotoxic compounds responsible for the observed toxicological activity. These results recommended further investigation of plant’s cytotoxic potential using *in vitro* anticancer cell lines.

**Table 5:** Cytotoxicity and *Streptomyces* hyphae formation inhibition potential of different solvent extracts of leaf, stem and fruit of *D. innoxia*. Values are presented as mean ± standard deviation of triplicate analysis

Extracts	Brine shrimp cytotoxicity (Concentration μg/ml)		THP-1 cytotoxicity (Concentration μg/ml)		Protein kinase inhibition	
	% mortality	LC_50_	% mortality	IC_50_	Diameter (mm ± SD)	
	1000		10		Clear zone	Bald zone
Leaf						
Nh	100.0 ± 0.00^*a*^	235.44 ± 1.12	22.32 ± 2.41^*c*^	˃10	--	7 ± 0.38
C	90.00 ± 0.49^*b*^	199.91 ± 0.98	80.95 ± 1.77^*a*^	5.91 ± 0.78	13 ± 0.18^*b*^	21 ± 0.29
A	96.60 ± 0.94^*b*^	226.67 ± 1.34	18.22 ± 1.68	˃10	7 ± 0.15	11 ± 0.47
EthA	98.30 ± 0.47	150.48 ± 1.14	23.22 ± 2.13^*b*^	˃10	7 ± 0.33	11 ± 0.39
Eth	78.30 ± 1.25	133.26 ± 0.43	31.24 ± 2.99	˃10	11 ± 0.43	22 ± 0.47^*a*^
EC	90.0 ± 0.94^*c*^	154.90 ± 0.22	55.23 ± 3.22	8.9 ± 0.53	14 ± .41^*b*^	21 ± 0.52^*b*^
MC	81.60 ± 1.70^*a*^	85.94 ± 0.16	52.38 ± 2.87	9.8 ± 0.15	7 ± 0.32	10 ± 0.21
EthE	86.60 ± 0.94	186.95 ± 0.17	16.33 ± 3.18	˃10	7 ± 0.62	10 ± 0.15
MEth	88.30 ± 2.36	117.69 ± 0.18	18.32 ± 4.11	˃10	12 ± 0.51	18 ± 0.41^*b*^
E	78.30 ± 0.94^*a*^	97.08 ± 1.35	25.34 ± 1.45^*b*^	˃10	7 ± 0.13	9 ± 0.42
M	85.00 ± 1.25	97.06 ± 1.18	16.21 ± 2.45	˃10	6 ± 0.22^*b*^	9 ± 0.35
D	67.00 ± 1.70	247.27 ± 0.74	12.85 ± 0.83^*a*^	˃10	--	--
Stem						
Nh	100.45 ± 0.00^*a*^	194.95 ± 0.34	--	--	6 ± 0.44	--
C	90.50 ± 0.00^*a*^	125.53 ± 0.22	--	--	6 ± 0.58	--
A	96.60 ± 0.94^*b*^	155.33 ± 0.98	--	--	7 ± 0.42	13 ± 0.42^*b*^
EthA	98.35 ± 0.58^*b*^	153.38 ± 0.54	--	--	7 ± 0.22^*a*^	11 ± 0.22^*a*^
Eth	78.35 ± 0.82^*b*^	250.00 ± 0.34	--	--	9 ± 0.35^*a*^	18 ± 0.35^*a*^
EC	90.45 ± 0.94	154.90 ± 0.16	--	--	6 ± 0.28	9 ± 0.28^*a*^
MC	85.40 ± 1.25^*c*^	97.06 ± 0.17	--	--	11 ± 0.21^*a*^	22 ± 0.21^*a*^
EthE	86.60 ± 0.47^*b*^	117.69 ± 0.16	--	--	6 ± 0.45^*b*^	8 ± 0.45
MEth	88.30 ± 0.95^*b*^	117.69 ± 1.46	--	--	6 ± 0.27	9 ± 0.27
E	78.30 ± 0.47^*a*^	137.29 ± 1.65	--	--	8 ± 0.33	13 ± 0.33^*b*^
M	81.60 ± 0.82^*b*^	85.94 ± 0.76	--	--	9 ± 0.51^*b*^	14 ± 0.51^*c*^
D	65.56 ± 0.82^*b*^	250.00 ± 0.17	--	--	6 ± 0.47	8 ± 0.47
Fruit						
Nh	78.30 ± 1.25^*a*^	359.12 ± 0.18	76.19 ± 1.92^*a*^	3.49 ± 0.17	--	10 ± 0.81^*b*^
C	91.60 ± 1.25^*a*^	141.68 ± 1.34	76.66 ± 2.22^*a*^	4.52 ± 1.23	--	10 ± 0.54
A	93.30 ± 0.47^*a*^	240.91 ± 1.56	16.23 ± 2.23	˃10	11 ± 0.23^*b*^	18 ± 0.54^*a*^
EthA	91.60 ± 1.25^*b*^	114.40 ± 0.78	16.88 ± 2.23	˃10	11 ± 0.47^*b*^	17 ± 0.22^*a*^
Eth	93.30 ± 1.25^*a*^	201.96 ± 0.17	10.32 ± 1.45	˃10	--	10 ± 0.22
EC	91.65 ± 1.25^*a*^	234.92 ± 0.27	18.3 ± 2.55^*b*^	˃10	--	10 ± 0.24
MC	85.00 ± 2.83^*b*^	54.07 ± 0.16	18.3 ± 2.66^*a*^	˃10	--	9 ± 0.32
EthE	98.35 ± 0.47^*a*^	218.64 ± 0.34	61.91 ± 1.22^*b*^	8.88 ± 1.16	8 ± 0.38^*b*^	14 ± 0.38^*a*^
MEth	90.00 ± 1.63^*a*^	152.61 ± 1.14	15.62 ± 1.87	˃10	--	13 ± 0.62^*b*^
E	90.00 ± 0.82^*a*^	229.21 ± 1.56	18.23 ± 1.27	˃10	11 ± 0.22^*a*^	20 ± 0.67
M	85.00 ± 1.41^*b*^	164.39 ± 1.78	16.57 ± 3.24	˃10	7 ± 0.43^*c*^	10 ± 0.28^*a*^
D	65.00 ± 0.82	351.71 ± 1.76	10.34 ± 1.28	˃10	--	7 ± 0.56
DMSO	--	--	--	--	--	

Initially, the samples were evaluated at single highest concentration and the samples which showed more than 50 % inhibition/significant activity were tested at lower concentrations to find their LC_50_. Negative control: DMSO. LC_50_ of Doxorubicin (positive control employed in the brine shrimp lethality assay) was 5.93 μg/ml. IC_50_ of 5-Florouracil (5FU) and Vincristine (positive controls employed in anticancer assay) was 5 and 8.1 μg/ml respectively. Growth inhibition zone exhibited by surfactin (positive control in Protein kinase inhibition assay) was 20 ± 1.02 mm (bald zone)

Values (mean ± SD) are average of three samples of each plant extract, analyzed individually in triplicate (*n* = 1 × 3). ^a-c^ Means difference is highly significant, slightly significant, significant at *p* < 0.05

#### Cytotoxicity against THP-1 human leukemia cell line

The incidence of several cancers has increased exponentially with age from the fourth to eighth decade of life. Over 6 million people decease due to cancer worldwide each year, being the largest single cause of death in both men and women [17]. Keeping in view the prodigious cytotoxic potential as discovered through brine shrimp lethality assay; the plant extracts were further screened for an *in-vitro* cytotoxic activity using human leukemia (THP-1 ATCC# TIB-202) cell line (Table 5). Among all the leaf extracts, the chloroform extract was most potent as it considerably inhibited the cell line proliferation exhibiting 80.95 ± 1.77 % cell mortality at 10 μg/ml concentration and an LC_50_ 5.91 μg/ml. In our present study the stem extracts did not display any anticancer potential while in case of fruit, the most prominent lethality was shown by the chloroform and n-hexane extracts with an LC_50_ 4.52 and 3.49 μg/ml respectively which is comparable to the standard drugs 5-florouracil and vincristine with 50 % lethality at 5 μg/ml and 8.1 μg/ml respectively. Recently a new withanolide, dinoxin B was isolated from a methanol extract of *D. innoxia* leaves exhibited sub-micromolar LC_50_ values against multiple human cancer cell lines [4]. This is by far the first report (to the best of our knowledge) highlighting the anticancer proficiency of *D. innoxia* fruit phytochemicals, which according to all the previous studies have been reported in its leaves only.

#### Protein kinase inhibition assay

The results of protein kinase inhibition zones recorded for the test samples are presented in Table 5. Among all the extracts, a noteworthy inhibition zone of 22 mm clear, 11 mm bald phenotype was formed around the ethyl acetate extract of both leaf and stem, while the most prominent hyphae formation inhibition in case of fruit was presented by the ethanol extract (20 mm clear, 11 mm bald) lagged by acetone (18 mm clear, 11 mm bald) and acetone-ethyl acetate extract (17 mm clear, 11 mm bald). The non-toxic effect of DMSO (negative control) was confirmed by the absence of growth inhibition zone whereas surfactin, the positive control established a 16 mm bald growth inhibition zone. The results of the present study suggest that a moderately polar extraction solvent would be suitable for the extraction of phytoconstituents of *D. innoxia* that may >serve as a promising kinase inhibitory target while the extremes of extraction solvent polarities exhibited little or no activity. In the recent years, there has been a significant surge for the development of inhibitors of protein kinases from natural products especially plants. Protein phosphorylation at serine/threonine and tyrosine residues by protein kinases is one of the major regulatory mechanisms in biological processes including apoptosis, cell proliferation, cell differentiation, and metabolism. Deregulated phosphorylation at serine/threonine and tyrosine residues by protein kinases produced as a result of genetic alterations acquired early in tumorigenesis are often the cause of cancer. In this respect, inhibition of protein kinases has emerged as a promising target for cancer treatment (Yao et al. [12]). Protein kinase activity is critical for the aerial hyphae formation of *Streptomyces* and this prerequisite has been exploited in the present study to bioprospect the extracts for their kinase inhibitory activity so that their anticancer potential could be assessed. Using *Streptomyces* 85E as an assay strain for kinase inhibitors appears to identify a wide range of eukaryotic kinase modulators, presumably because the *Streptomycete* enzymes are evolutionary forerunners of their highly specific eukaryotic counterparts. An advantage of the whole cell *Streptomycete* assay is that it readily identifies cytotoxic activity of the compounds being tested. This simple assay permits the identification of signal transduction inhibitors for a variety of applications including anti-infective, antitumor agents and several of the inhibitors of mycobacteria [22].

### Antimicrobial assays

#### Antibacterial assay

Predictions of antibacterial activity in herbal compounds extracted from plant parts depend largely on plant part, solvent used for extraction and the organism tested [14]. Table 6 shows the antibacterial activity in terms of zone of inhibition (mm diameter) of various solvent extracts from different parts (leaves, stem and fruit) of *D. innoxia*. In our study the extracts producing a growth inhibitory zone ≥ 10 mm in agar disc diffusion assay were considered active and were further evaluated for MIC determination through broth micro dilution method. All the extracts showed better antibacterial activity against Gram-positive bacteria as compared to Gram-negatives. Among all the extracts 39 % of leaf, 16 % of stem and 29 % of fruit extracts were found to be active (zone ≥ 10 mm). Among all the bacterial strains tested, *Mirocococcus luteus* was found most susceptible with maximum inhibition by the n-hexane fruit extract producing zone of inhibition of 24 mm (MIC = 3.70 μg/ml). Data indicated that extracts prepared from leaves possess better antibacterial activity than those prepared from stem and fruit. Among all the leaf extracts a maximum zone of growth inhibition was displayed by the ethyl acetate, ethanol and acetone extracts against *K. pneumonae, S. typhi, M. luteus* and *S. aureus* respectively. Among all the stem extracts the maximum growth inhibition zone of 20 mm was produced by the ethanolic extract having MIC of 3.70 μg/ml. The n-hexane fruit extract presented antibacterial activity with an inhibition zone ranging between 7 and 24 mm. The absence of growth inhibition zones confirmed the non-toxic effect of DMSO (negative control) while cefixime served as positive control. Hydroxylated phenolic compounds such as caffeic acid and catechol from various plant extracts have shown to be toxic to microorganisms The mechanisms thought to be responsible for phenolic toxicity to microorganisms include enzyme inhibition by the oxidized compounds, possibly through reaction with sulfhydryl groups or through more nonspecific interactions with the proteins [23]. In our present study the antimicrobial activity exhibited by various extracts might be due to these hydroxylated phenols which are also quantified through HPLC.

**Table 6:** Antibacterial activity of leaf, stem and fruit extracts of *D. innoxia*

Extracts	^a^Diameter of growth inhibition zone (mm ± SD)							
	*K. pneumoniae*	MIC (μg/ml)	*S. typhimurium*	MIC (μg/ml)	*M. luteus*	MIC (μg/ml)	*S. aureus*	MIC (μg/ml)
Leaf								
Nh	8.5 ± 0.76^d^	--	10 ± 2.85^b^	33.33	7 ± 1.84^d^	--	9 ± 1.89^c^	--
C	10 ± 0.96^c^	33.33	6.5 ± 1.32^b^	--	9 ± 1.45^d^	--	9 ± 2.31^c^	--
A	10 ± 0.76^c^	33.33	7.5 ± 1.33^d^	--	14.5 ± 1.1^b^	3.70	10 ± 1.63^b^	100
EthA	10 ± 0.98^c^	100	7 ± 1.54^d^	--	12 ± 1.17^c^	33.33	9 ± 1.78^c^	--
Eth	12 ± 0.22^c^	100	8 ± 1.24^c^	--	18 ± 1.34^b^	3.70	8 ± 1.49^d^	--
EC	10 ± 0.48^c^	100	7.5 ± 1.34^d^	--	12 ± 1.22^b^	33.33	8.5 ± 1.87^d^	--
MC	7 ± 0.45^d^	--	6 ± 2.21^d^	--	16 ± 1.45^b^	3.70	6 ± 1.89^d^	--
EthE	12 ± 0.18^b^	33.33	7.5 ± 1.76^d^	--	14 ± 1.45^c^	33.33	7 ± 2.21^d^	--
Meth	9.5 ± 0.34^c^	--	7 ± 1.32^d^	--	10 ± 1.34^c^	100.00	8. 5 ± 1.38^d^	--
E	8 ± 0.67^d^	--	17 ± 1.45^d^	11.11	13 ± 1.45^c^	11.11	9.5 ± 1.22^b^	--
M	7 ± 0.34^d^	--	8 ± 1.24^c^	--	16 ± 1.67^b^	3.70	7 ± 1.23^d^	--
D	7 ± 0.56^d^	--	7 ± 1.23^d^	--	14 ± 1.13^c^	11.11	6 ± 1.43^d^	--
Stem								
Nh	--	--	10 ± 1.67^c^	33.33	8 ± 2.34^c^	--	10 ± 1.83^b^	100
C	--	--	14 ± 0.88^b^	11.11	8 ± 1.45^c^	--	9 ± 1.21^c^	--
A	--	--	7 ± 1.21^d^	--	8 ± 2.2^b^	--	--	--
EthA	--	--	10 ± 2.67^c^	100	7 ± 1.22^d^	--	--	--
Eth	--	--	7 ± 2.34^d^	--	--	--	--	--
EC	--	--	7 ± 1.23^d^	--	13 ± 1.3^b^	100	--	--
MC	9 ± 1.87^c^	--	9 ± 1.56^c^	--	--	--	7 ± 2.21^d^	--
EthE	8 ± 2.14^c^	--	--		7 ± 1.45^d^	--	--	--
Meth	--	--	--		7 ± 0.77^d^	--	--	--
E	--	--	10 ± 2.13^c^	100	20 ± 1.82^c^	3.70	--	--
M	9 ± 2.32^c^	--	6 ± 1.43^d^	--	7 ± 1.23^d^	--	8 ± 1.21^c^	--
D	--	--	10 ± 1.56^c^	100	0	--	--	--
Fruit								
Nh	--	--	12 ± 1.21^b^	100	24 ± 1.32^b^	3.70	--	--
C	8 ± 1.23^c^	--	8 ± 2.45^d^	--	9 ± 1.22^c^	--	9 ± 1.32^d^	--
A	8 ± 1.45^c^	--	7 ± 1.13^d^	--	8 ± 1.45^d^	--	--	--
EthA	--	--	13 ± 2.21^b^	33.33	10 ± 3.12^c^	100	10 ± 1.21^c^	100
EC	--	--	8 ± 1.56^d^	--	--	--	--	--
MC	--	--	10 ± 1.56^c^	100	9 ± 1.82^c^	--	--	--
EthE	--	--	11 ± 1.65^c^	100	8 ± 1.21^d^	--	--	--
Meth	9 ± 1.76^c^	--	10 ± 1.67^c^	100	8 ± 2.13^d^	--	--	--
E	10 ± 1.12^b^	100	12 ± 2.21^b^	100	8 ± 1.23^d^	--	12 ± 1.28^b^	33.33
M	7 ± 1.23^d^	--	12 ± 1.54^b^	33.33	9 ± 1.51^b^	--	--	--
D	--	--	--	--	10 ± 1.21^c^	100	--	--
DMSO	--	--	--	--	--	--	--	--
Cefixime	28 ± 0.07	3.33	26 ± 0.21	3.33	18 ± 0.44	1.11	16 ± 1.22	1.11

^a^Zone of inhibition including the diameter of disc (5 mm). In each disc, the sample size was 100 μg per disc (5 μl) in disc diffusion assay. Values (mean ± SD) are average of three samples of each plant extract, analyzed individually in triplicate (*n* = 1 × 3). ^b-d^ Means difference is highly significant, slightly significant, and significant at *p* < 0.05. --: No activity in disc diffusion assay or not active (zone ≥ 10 mm) for MIC determination

#### Antifungal assay

The plant’s antifungal potential was assessed against four strains of filamentous fungi. The antifungal growth inhibitory activities of extracts of various plant parts are summarized in Table 7. The data indicate that acetone extract of leaf and stem while n-hexane extract of fruit showed a prominent growth inhibition zone of 22, 20 and 24 mm against *A. niger* respectively. Among all the extracts, lowest minimum inhibitory concentration (MIC) of 12.5 μg/ml against *A. niger* was presented by the n-hexane fruit extract. It has been reported that *A. niger* produces potent mycotoxins on foodstuffs and is the most prevalent fungus affecting corn [24]. A moderate antifungal activity was shown by almost all the extracts against *Mucor sp*. with an average diameter of growth inhibition zone ranging between 7 and 14 mm. It was observed that mostly the antifungal activity increased as the polarity decreased. Thus the chloroform and n-hexane extracts showed better antifungal activity than aqueous or ethanolic extracts. The absence of growth inhibition zones confirmed the non-toxic effect of DMSO whereas standard drug Clotrimazole exhibited maximum activity (30 ± 1.54 mm). Well-known plant secondary metabolites exhibiting antifungal activity include flavonoids, phenols and phenolic glycosides, unsaturated lactones, sulphur compounds, saponins, cyanogenic glycosides and glucosinolates [24]. Therefore in our present study the antifungal activity might be due to the phenolic compounds such as flavonoids.

**Table 7:** Antifungal activity of leaf, stem and fruit extracts of *D. innoxia* tested against filamentous fungi

Extracts	^a^Diameter of growth inhibition zone (mm ± SD)							
	*A. fumigatus*	MIC μg/ml	*Mucor sp*.	MIC μg/ml	*A. niger*	MIC μg/ml	*A. flavus*	MIC μg/ml
Leaf								
Nh	8 ± 3.24^c^	100	10 ± 2.85^b^	100	7 ± 2.21^d^	100	--	--
C	8 ± 1.43^c^	100	--	--	9 ± 1.32^d^	100	--	--
A	7 ± 1.56^d^	100	8 ± 1.34^c^	100	22 ± 1.5^b^	25	--	--
EthA	7 ± 2.45^d^	100	12 ± 3.45^b^	50	10 ± 1.78^c^	--	--	--
Eth	--	--	7 ± 1.67^d^	100	--	--	--	--
EC	--	--	--	--	--	--	--	--
MC	--	--	--	--	--	--	--	--
EthE	7 ± 1.22^d^	100	10 ± 3.27^b^	100	--	--	--	--
Meth	--	--	9 ± 1.65^c^	100	--	--	--	--
E	--	--	7 ± 2.43^d^	100	10 ± 1.45^c^	100	--	--
M	--	--	9 ± 2.56^c^	100	7 ± 1.67^d^	100	--	--
D	--	--	8 ± 1.54^c^	100	--		--	--
Stem								
Nh	--	--	10 ± 1.67^c^	50	8 ± 2.34^c^	100	10 ± 1.8^c^	100
C	--	--	14 ± 0.88^b^	50	8 ± 1.45^c^	100	9 ± 1.21^c^	100
A	--	--	7 ± 1.21^d^	100	20 ± 2.23^b^	25	--	
EthA	--	--	10 ± 2.67^c^	50	7 ± 1.22^d^	100	--	
Eth	--	--	7 ± 2.34^d^	100	--		--	
EC	--	--	7 ± 1.23^d^	100	13 ± 1.32^c^	100	--	
MC	9 ± 1.87^c^	100	9 ± 1.56^c^	100	--		7 ± 2.21^d^	100
EthE	8 ± 2.14^d^	100	--		7 ± 1.45^d^	100	--	
MEth	--	--	--		7 ± 0.77^d^	100	--	
E	--	--	10 ± 2.13^c^	100	8 ± 1.82^d^	100	--	
M	9 ± 2.32^c^	100	6 ± 1.43^d^	100	7 ± 1.23^d^	100	8 ± 1.21^c^	100
D	--	--	10 ± 1.56^c^	100	--		--	
Fruit								
Nh	--	--	12 ± 1.21	100	24 ± 1.32^b^	12.5	--	--
C	8 ± 1.23^d^	100	8 ± 2.45^d^	100	9 ± 1.22^c^	100	9 ± 1.32^c^	100
A	8 ± 1.45^d^	100	7 ± 1.13^d^	100	8 ± 1.45^d^	100	--	--
EthA	--	--	13 ± 2.21	50	10 ± 3.12^b^	100	10 ± 1.21^b^	100
Eth	--	--	--	--	16 ± 1.67^b^	12.5	--	--
EC	--	--	8 ± 1.56^d^	100	--	--	--	--
MC	--	--	10 ± 1.56^c^	50	9 ± 1.82^c^	100	--	--
EthE	--	--	11 ± 1.65^c^	100	8 ± 1.21^d^	100	--	--
MEth	9 ± 1.76^c^	100	10 ± 1.67^c^	100	8 ± 2.13^d^	100	--	--
E	10 ± 1.12^c^	100	12 ± 2.21	50	8 ± 1.23^d^	100	12 ± 1.28^b^	100
M	7 ± 1.23^d^	100	12 ± 1.54	50	9 ± 1.51^c^	100	--	--
D	--	--	--	--	10 ± 1.21^b^	100	--	--

^a^Zone of inhibition including the diameter of disc (5 mm). In each disc, the sample size was 100 μg per disc (5 μl) in disc diffusion assay. Values (mean ± SD) are average of three samples of each plant extract, analyzed individually in triplicate (*n* = 1 × 3). ^b-d^ Means difference is highly significant, slightly significant, and significant at *p* < 0.05. --: No activity in disc diffusion assay or not active (zone ≥ 10 mm) for MIC determination

## Conclusions

The antioxidant, antimicrobial and cytotoxic activities of various plant parts of *D. innoxia* reported in this study, may explain some of the traditional medicinal uses of this plant. The use of a wide-ranging solvent system polarity resulted in a complete phytochemical and biological profiling of different plant parts. The current study proposes that ethyl acetate extract of *D. innoxia* may be a potential source of phytochemicals inciting substantial antioxidant capability, tumorigenic kinase inhibitors and antibacterial compounds. Similarly chloroform soluble extract of this plant is remarkably effective against brine shrimps and in the inhibition of THP-1 human leukemia cell line proliferation which signifies its cytotoxic potential. These activities could be important in relation to finding out its unexplored efficacy and can be a potential source of chemically interesting and biologically important anticancer drug candidates. The present study calls for further research aimed at isolating the bioactive compounds responsible for the observed activity. These compounds could serve as novel scaffolds in search for new drugs. Further investigations involving bioassay guided isolation of these crude extracts is recommended.

## Abbreviations

DMSO: Dimethyl sulfoxide
TPC: Total phenolic contents
TFC: Total flavonoid contents
DPPH: 2, 2-diphenyl-1-picrylhydrazyl
RSA: Radical scavenging activity
IC_50_: 50 % inhibitory concentration
TRP: Total reducing power
TAC: Total antioxidant capacity
MIC: Minimum inhibitory concentration
LC_50_: Lethal concentration causing 50 % mortality
ZOI: Zone of inhibition
